# Heat transfer intensification in an actuated heat exchanger submitted to an imposed pressure drop

**DOI:** 10.1371/journal.pone.0219441

**Published:** 2019-07-11

**Authors:** Kevin Schmidmayer, Prashant Kumar, Pascal Lavieille, Marc Miscevic, Frédéric Topin

**Affiliations:** 1 Aix-Marseille University, Marseille, France; 2 LAPLACE, University of Toulouse, Toulouse, France; University of New South Wales, AUSTRALIA

## Abstract

This paper is dedicated to the analysis of the improvement of heat transfer and the reduction of the pressure losses induced by the use of an active exchanger of millimeter size in a cooling loop. For pressure conditions imposed at the terminals of such a mini-channel whose upper wall is deformed by a progressive sinusoidal wave and for low Reynolds numbers (*Re* < 1000), we study the influence of the deformation parameters on the thermo-hydraulic performance of the exchanger (flow, heat transfer). The mechanical power applied to the deformed wall is connected to these parameters as well as to the pressure difference imposed by the external pump. The overall performance increases slightly with the value of the mechanical power up to a critical value for a given wall corrugation. Nevertheless, overall performance is up to 2 orders of magnitude higher than conventional static corrugated channels.

## Introduction

At small scale (e.g. millimeter scale), the temperature control and associated heat flux management are crucial in many applications: microelectronics, embedded or fixed power electronics systems, power station, air conditioners, heat pumps, thermal processes in the fields of industrial metallurgy, chemistry, food, etc. During the last two decades, laminar flow in channels with corrugated walls has been intensively investigated, both experimentally and numerically. Wall corrugation has attracted particular interest in applications where efficiency of mixing processes is of fundamental importance because of its relative technological simplicity. Corrugated walls allow increasing turbulence and thus lead to better heat transfer coefficients, thereby increasing overall thermal performance as compared to flat heat exchanger.

Many studies on the influence of the corrugations’ shape (e.g. sinusoidal, triangular, square, trapezoidal) in plate heat exchangers have been conducted both experimentally and numerically. Among these studies, corrugations have been applied to either the two walls or only one of the walls. The impact of geometrical parameters such as corrugation angle and aspect ratio (defined as the ratio between the corrugation height and width) has also been widely investigated. For instance, Nishimura et al. have studied heat transfer and pressure drop in corrugated channels, either sinusoidal [1] or arc-shaped [2]. They concluded that the critical Reynolds number depends on the corrugation shape and that laminar to turbulent flow transition occurs at lower *Re* than for straight channels (*Re* = 300, for sinusoidal channels). This was attributed to the development of unsteady vortex motion. Niceno and Nobile [3] have conducted an extended numerical study considering the two same shapes of corrugation than the previous ones. They showed both steady and time-dependent (but not turbulent) flows at moderate Reynolds numbers. The threshold Reynolds number for the unsteady regime depend on the corrugation shapes, namely *Re* = 60 − 80 for arc-shaped channel and *Re* = 175 − 200 for sinusoidal channel. The induced self-oscillations in unsteady regime increase significantly (up to a factor 3) the heat transfer rate. This increase was more pronounced for arc-shaped channel, but the friction factor was augmented in a proportional manner.

The heat transfer enhancement in corrugated channel has also been demonstrated in experimental studies. For example, Gradeck et al. [4] performed experiments in corrugated channels to study effects of hydrodynamic conditions on the enhancement of heat transfer for single-phase flow. The local temperature measurements were used to evaluate the local and global heat transfer coefficients for a wide range of Reynolds numbers (0 < *Re* < 7500). The mixing effect induced by the recirculation in the wake of the corrugations always conducts to higher heat transfer than the one obtained with a flat channel. Islamoglu and Parmaksizoglu [5] examined the effect of corrugation height on heat transfer and friction characteristics in a corrugated channel made of two parallel walls, each of them having a saw-tooth shape. The analyses were conducted for a single corrugation angle of 20° and for Reynolds numbers from 1200 to 4000. In accordance with the studies cited above, they obtained a significant increase in heat transfer (enhancement of the Nusselt number from 15 to 75%), this increase being higher when the channel height is high. Nevertheless, the friction factors increase along with the corrugation height. So, defining the performance of the corrugation as the ratio between Colburn factor and friction factor, the best corrugated channel was found to be the one with the smallest height.

In addition to Reynolds number and non-stationary effects, geometrical parameters of the corrugation, such as wavelength and amplitude, or the geometry of each corrugation, can have a strong effect on thermo-hydraulic behavior. Wang and Chen [6] numerically studied the impact of the amplitude to wavelength ratio on the performance in the case of forced convection flow. Thermo-hydraulic performance was evaluated considering a periodic array of corrugations. It was found that the corrugated channel is very effective for large amplitude to wavelength ratio when Reynolds number is quite high. Naphon [7] performed experiments and numerical simulations to characterize heat transfer and pressure drop characteristics in a channel with double V-corrugated surfaces. The corrugation angle was varied from 0° to 60° corresponding to an increase of the number of wavelengths for a given channel length (i.e. increase in surface area) while keeping the same average channel height. The author then extended his work to others corrugation shapes [8], including the effect of the phase-shift between upper wall and lower wall (i.e. parallel or staggered corrugations). He concluded that breaks and destabilizations occurring in the thermal boundary layer as the fluid flows through the corrugated surfaces have significant effects on the enhancement of heat transfer and pressure drop. Elshafei et al. [9] experimentally studied heat transfer and pressure drop in corrugated channels with uniform wall temperature considering different cases of spacing and phase shifting between corrugations of the upper wall and corrugations of the lower wall. Their results showed that the variation in phase shift has no significant effect on the friction factor compared to the effect of spacing variation, especially for high Reynolds numbers.

As the characteristic dimension may also affect the heat transfer behavior, Liu et al. [10] conducted a numerical study of forced convection heat transfer occurring in small channel (corrugation amplitude of 1mm). Different corrugations were referenced: semicircular structure as ridge-shaped groove, triangular as V-shaped groove, tilted V-shape as shield-shaped groove, rectangular as straight slot groove and plain surface as flat channel. These authors pointed out that the structure of the micro-channel is especially important on heat transfer characteristics and obtained about 1.3 times higher Nusselt number for microstructure with shield-shaped groove compared to that of plain surface. So, the use of corrugation or other static geometrical singularities makes it possible to significantly improve the heat transfer due to the disturbance induced by the boundary layers and the appearance of transient behaviors. Most studies have shown an increase in overall thermal performance (up to 5 times) depending on the geometry of the corrugation. Nevertheless, this also induces a significant increase in pressure losses which is prohibitive for many applications, particularly for small diameter systems. The same trend is also observed for microchannels with rough surfaces, for relative height below 10% the impact on heat transfer remains however limited (less than few%) while the pressure drop increase is more significant [11].

During the last decade, with the development of miniaturized electromechanical actuation systems the impact of active elements on heat transfer has been widely studied (see review in [12]). For example, Ma et al. [13] and Li et al. [14] used piezoelectric vibrating blades to disrupt the thermal boundary layer and reduce the pressure drop. Leal et al. [15] appear to be the first study to introduce a dynamic wall in such mini-channels within heat exchanger. They proposed a new concept of heat exchanger on a millimeter scale. It consists in dynamically deforming at least one of its walls by a progressive wave to create an active corrugated channel. They obtain, with low Reynolds number, a strong intensification of heat transfer associated with a pumping effect. Schmidmayer et al. [16] performed a numerical analysis of the behavior of such a device inserted in a liquid loop composed only with passive components (i.e. the pumping of the fluid within the loop is performed only by the actuated heat exchanger). They developed a virtual prototype of such an active system and processed to systematic studies of the influence of the deformation parameters on heat transfer and pumped flow. They conclude that this type of system has interesting characteristics for the targeted applications:

- The overall performance of the proposed system is always significantly better than the classical static channels.
- A strong intensification of heat transfer is obtained, even for highly degraded waveforms compared to sinusoidal progressive waves.
- a significant pumping effect is highlighted in all cases but with a degradation related to that of the waveform. The mechanical power required for the operation of the system is proportional to the desired input-output pressure difference, the waveform, and its amplitude.

In the present work we extended these two last studies to the association of the active heat exchanger with an external pumping device in a cooling loop and explore the impact of various operating parameters on thermo-hydraulic characteristics using a realistic geometrical model. Parametric studies are presented for straight as well as static and dynamic corrugated channels, both from local and global points of view for various imposed pressure differences across the channel.

## Virtual prototype

**Fig 1:**
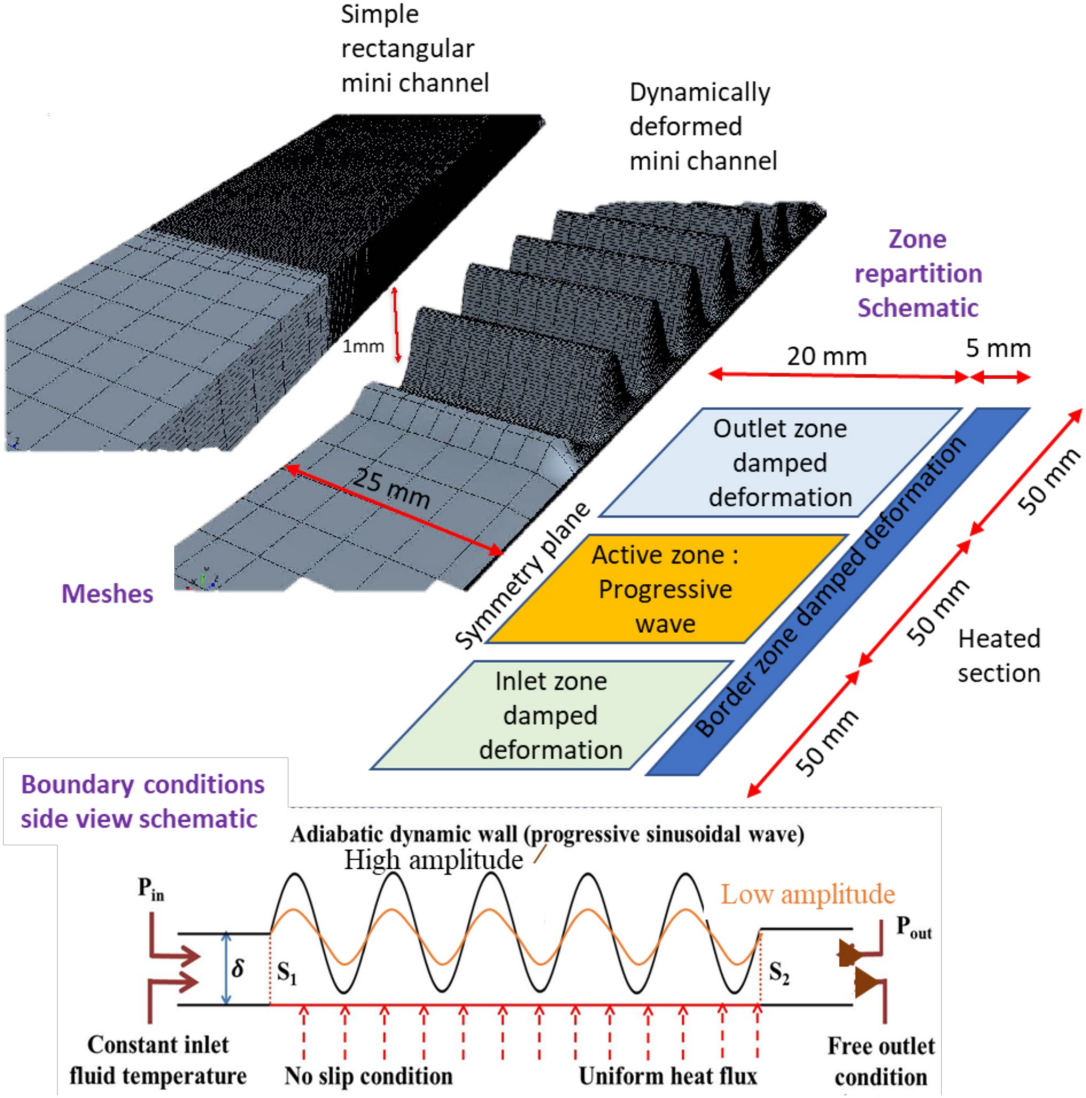
Geometrical and numerical configurations. Top: View of one of the used meshes. The same locations and point of view are kept for both channels. Left: Straight channel (Height 1mm). Right Corrugated channel (Amplitude *A*_0_ = 90%). Vertical scale is magnified 100 times. Center: Top view schematic of the half-channel model. The vertical plane passing through the central axis of the channel is a plane of symmetry, so only half of the channel was simulated (W/2 = 25mm). Bottom: Side view schematic of the DDMC. The lower wall was fixed and subjected to a constant and uniform heat flux over the imprint of heating zone length (length, L: 50 mm, imposed heat power Γ: 125 W) facing the actuated upper wall. A negative pressure difference (Δ*P*_*s*_ = *P*_*out*_ − *P*_*in*_ < 0) is imposed between outlet and inlet sections of the channel, deformed membrane drawn for 2 amplitudes (black and orange).

In order to study fluid flow and heat transfer characteristics in small-scale heat exchanger, we use here a virtual prototype derived from the one previously developed in [16]. Such a dynamically deformed mini-channel (DDMC) allows us gaining a better understanding of heat transfer and flow behavior as well as the various operating parameters influences. In the present configuration (Fig 1) the upper wall and the sidewalls are adiabatic. A no-slip condition is imposed on all the physical walls. The height (*δ*) of the channel, defined as the average distance between the top and bottom walls, is 1 mm while the width (W) of the channel is 50 mm. The main differences with the virtual prototype developed in [16] are the prescribed boundary conditions and marginal adjustments of mesh dimensions and time step management used to improve CPU consumption and overall convergence behavior linked to the software improvement along time. The behavior of the DDMC is studied for different negative values of pressure differences between extremities (Δ*P*_*s*_ = *P*_*out*_ − *P*_*in*_ < 0). This configuration represents the case of a DDMC connected to a circuit comprising an external pump that generates this pressure difference as opposed to the situation previously studied in [16].

The upper wall (“membrane” in the following), is deformed to form a travelling sinusoidal wave. To simulate a realistic system, the membrane actuation amplitude is progressively damped along inlet and outlet zone whose extremities are fixed. Experimentally, this could be realized by clamping the periphery of a membrane on a base plate and actuating the center part of the membrane using several actuators, e.g. piezoelectric’s ones. Alternatively, a cames-shaft with multiple pistons could be used. In order to avoid the effects of transient reverse flow across boundaries, the lengths of inlet and outlet static sections have been chosen sufficiently long (50 mm) to ensure that no fluid from outside the modeled domain may enter the measurement zone during the eventual transient backflow stages. The displacement of the membrane is obtained using a three step procedure (see [16] or S1 Appendix for details):

- displacing the upper wall at chosen height *δ* and fixing it in inlet and outlet static zone;
- adding the movement of the membrane described by a progressive wave equation;
- damping the movement (interpolation) near lateral border and between actuated zone and inlet (resp. outlet) static zones.

Parametric studies were carried out for a given frequency (*f*_*r*_ = 50*Hz*) and wavelength (λ = 1*cm*), and for wave amplitude varying from 60% up to 98% of the channel height. The average height of the channel, i.e. the mean position of the membrane (*δ*), is kept constant. The difference in amplitude thus modifies the extremal distances between membrane and heated wall. The results presented in the following sections focus on the “active zone” namely between *S*_1_ and *S*_2_ sections (see Fig 1). All representative thermo-hydraulic quantities were calculated in this zone. The active region is finely meshed with refinement in the vicinity of the lateral walls while larger cells were used near the symmetry plane and along the static inlet and outlet regions (see Fig 1). Meshing such extremely thin shape (length -resp. width- to height ratio up to 2500) subjected to large amplitude deformations (up to 98% of channel height) is not simple. Indeed, the convergence and precision of the calculations need to be good to accurately describe the behavior of the dynamic transfer, but the computational time must be low enough in order to perform systematic studies. Preliminary test and previously published results have shown that the typical used mesh (Fig 1) present adequate shape quality index during the whole calculations is fine enough in the constriction and lead to reasonable anisotropy of the cells. The three-dimensional incompressible and transient conjugate flow and heat transfer problems described by the classical combination of continuity, momentum and energy equations were solved in dynamically deformed structures (see more detailed description in [15]) using CFD commercial software StarCCM+ considering constant or variable physical properties of the fluid as a function of temperature. We used water properties taken from IAPWS-97 formulation (either supposed constant and taken at 27°C) or temperature dependent. The numerical resolution used a segregated approach with implicit second order temporal discretization and optimized relaxations factors in order to obtain adequate convergence behavior [16]. The corrugation shape and fluid flow were first initialized for all studied cases. From preliminary tests and our earlier work analysis, a time step of 1/50 period is used to allow a precise description of all quantities. Then, calculations were conducted until a periodic stationary regime is reached. This latter point was checked by analyzing global heat transfer and flow characteristics. The calculations over a long time have been performed until an excellent estimation of the established conditions is obtained. Namely we calculated until all monitored quantities (principally temperatures, flow rate and heat transfer coefficient) varied by less than 1% between 2 consecutive periods. Finally, an additional time period was calculated to extract all instantaneous and time-averaged values of all physical quantities. A preliminary mesh convergence analysis was conducted to determine a mesh sufficiently coarse for use in systematic studies while conserving adequate precision. A fair convergence of the calculations was obtained for the various amplitudes for a sufficient number of meshes. In this case, the overall thermohydraulic properties measured remain almost constant, indicating correct convergence (Tables 1 and 2). Meshes composed of 245752 (straight channel) and 281072 (dynamic corrugated channel) cells were chosen to conduct the systematic studies presented in the following sections.

**Table 1:** Mesh convergence for the straight channel.

Mass flow rate (g/s)	Number of cells	Wall Temperature (K)	Heat transfer coefficient (*W*.*m*^−2^.*K*^−1^)
10	5240	322.71	2722
	66900	322.79	2715
	**245752**	**322.81**	**2714**
	485368	322.82	2714
2	5240	317.72	3305
	66900	317.83	3290
	**245752**	**317.85**	**3288**
	485368	317.86	3287

Global flow and heat transfer properties for different number of cells; bold font indicates chosen mesh to carry out systematic studies.

**Table 2:** Mesh convergence for the dynamic corrugated channel.

Amplitude	Number of cells	Mass flow rate (*g*.*s*^−1^)	Heat transfer coefficient (*W*.*m*^−2^.*K*^−1^)
60%	43560	11.1	4409
	159766	14.7	4382
	**281072**	**14.3**	**4350**
80%	43560	16.3	5985
	159766	21.0	6190
	**281072**	**20.4**	**6128**
95%	43560	-	-
	159766	26.6	10300
	**281072**	**25.6**	**11413**

Global flow and heat transfer properties for different mesh sizes and for Δ*P*_*s*_ = −50*Pa*; bold font indicates chosen mesh to carry out systematic studies.

## Straight channel: Global thermo-hydraulic results

The numerical tool was first used to study the thermo-hydraulic behavior of a simple reference case straight channel. Impact of the temperature dependence of the fluid thermo-physical properties on global thermo-hydraulic properties was first investigated. Further, the thermo-hydraulic results obtained numerically in straight channel were validated against experimental data reported in the work of Lee et al. [17].

**Fig 2:**
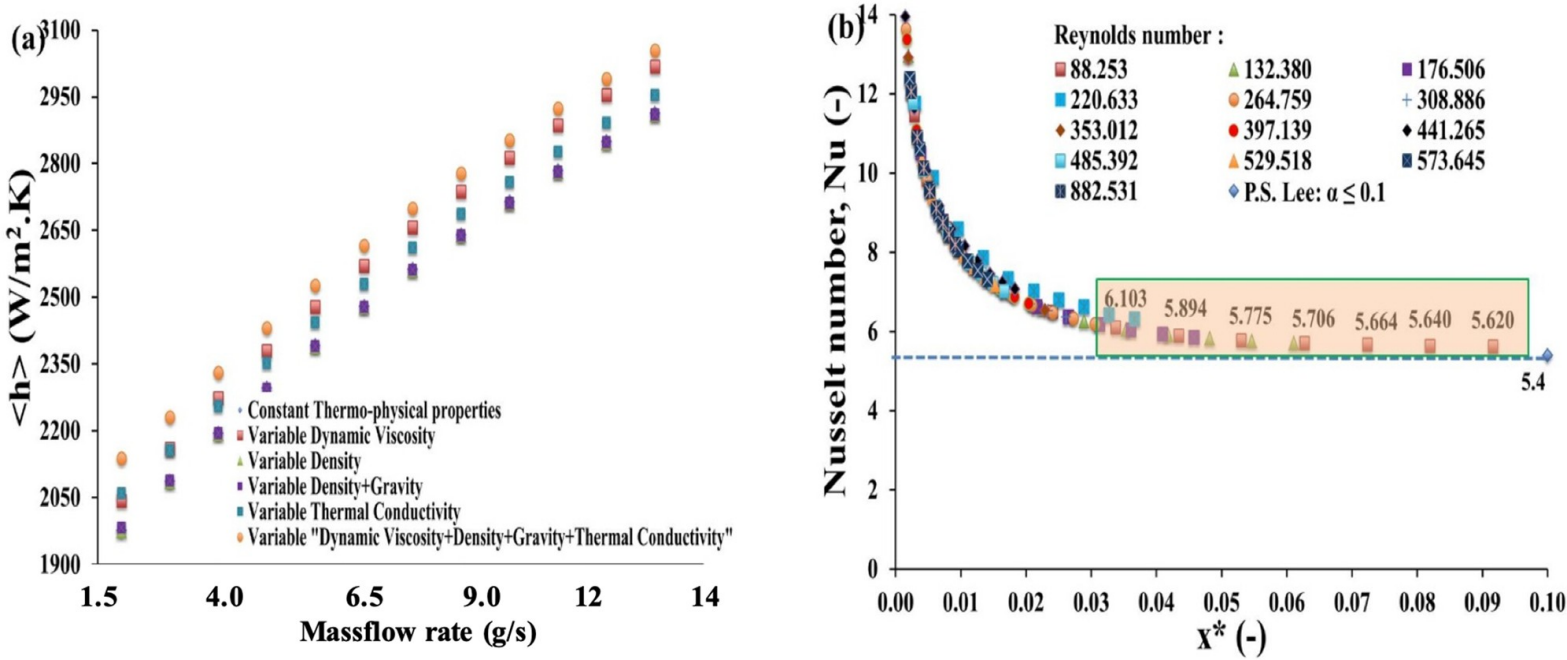
Impact of thermo-dependent fluid properties and comparison with analytical results. a) Global heat transfer coefficient (< *h* >) obtained using constant and variable thermo-physical properties as a function of the mass flow rate. (b) Evolution and validation of Nusselt number along x* for different Reynolds numbers in a rectangular channel heated by a constant heat flux on one side. Analytical results from Lee et al. [17] are obtained for an aspect ratio *α* ≤ 0.1.

In literature, most commonly, the fluid thermo-hydraulic properties dependence with temperature are neglected. In the present study, the fluid temperature difference between inlet and outlet sections varies between 10°C and 40°C depending on mass flow rates while the inlet fluid temperature was maintained at 27°C. Numerical simulations were carried out first for constant properties at mean water temperature. Further, temperature influence on properties of water (Faghri et al. [18]) was considered for several cases. Heat transfer coefficient as a function of mass flow rate for constant as well as variable thermo-physical properties of water is presented on Fig 2a.

Heat transfer coefficient is defined as:

$$\begin{array}{c} <h>=\frac{\Phi }{S(<T_{w}>-<T_{f}>)} \end{array}$$ (1)

where Φ is the total applied heat power, *S* is the surface area and < *T*_*w*_ > and < *T*_*f*_ > are the mean temperature of the wall and the fluid, respectively.

This heat transfer coefficient slightly differs (about 5%) if the thermo-physical properties of water are considered constant or variable. This indicates that for cases considered in this work (for which the inlet to outlet temperature difference remains moderate), the thermal dependence of physical properties can be neglected. Thus, thermo-hydraulic characteristics were calculated considering constant physical fluid properties (i.e. *ρ*, *μ*, *C*_*p*_, *k*_*f*_) in the following paragraph. Obviously if higher temperature variations are obtained or if the fluid properties variations are more important (e.g. oil) then the fluid properties variations will significantly change the flow pattern and thus the heat transfer behavior.

For our validation case (straight channel), current numerical results obtained using constant fluid properties at mean fluid temperature were compared to the experimental data of Lee et al. [17] for an aspect ratio *α* ≤ 0.1 in terms of Nusselt number (Nu) as a function of the dimensionless parameter (x*) as presented on Fig 2b, x* being defined as:

$$\begin{array}{c} x^{*}=\frac{x}{D_{h}RePr} \end{array}$$ (2)

where *D*_*h*_ is the hydraulic diameter, *Re* is the Reynolds number and *Pr* is the Prandtl number). Note that the aspect ratio of our straight channel is *α* = 0.02. Lee et al. [17] performed experiments with rectangular ducts heated on one of the sides by a constant and uniform heat flux while the other sides were kept adiabatic. These authors compared their results with different numerical (e.g. Wibulswas [19]) and analytical (e.g. Phillips [20]) data and observed a good agreement with a deviation varying from 2.4% to 8.1%. The difference between current numerical results and these experimental data when *α* ≤ 0.1 is extremely low. This excellent agreement for the straight channel allows us to extend and explore the thermo-hydraulic performances of dynamic wall corrugated channel in the following section.

## Dynamic corrugated channel: Thermo-hydraulic analysis

The heat transfer coefficient with constant as well as variable thermo-physical properties of the fluid were first compared in case of dynamic corrugated wall. We previously found that very low impact of fluid properties variations with temperature was obtained on performances of the dynamic heat exchanger in self-pumping regime [16]. Local viscosity variations only produce minor change as the flow pattern is mainly driven by the wall movement. This latter disrupts boundary layers leading to a homogeneous temperature in the fluid, thus limiting the properties variations. As this point is particularly interesting in terms of design rule for such system the same kind of analysis has also been conducted for the present case where the flow is mainly produced by a specific pumping device. As expected, the temperature dependence of the fluid properties does not significantly influence the values of mass flow rate (variation of about 1.5% compared to reference case) and heat transfer coefficient (less than 3% difference). As the global thermo-hydraulic performances were not influenced by the variations of the fluid properties, systematic studies were further conducted using constant fluid properties.

Characteristics of mass flow rate and heat transfer as a function of pressure differences between entrance and exit sections of the dynamic corrugated channel are then presented in the following sections. It can be noticed that there are not yet experimental data in the literature to validate the case of dynamic corrugated channel wall. Nevertheless, the present numerical results follow qualitatively the same trend than the work of Léal et al. [15]. This latter was developed for an active component in a simpler geometry and academic boundary conditions. This lends confidence to the thermo-hydraulic results detailed hereafter.

## Local analysis

**Fig 3:**
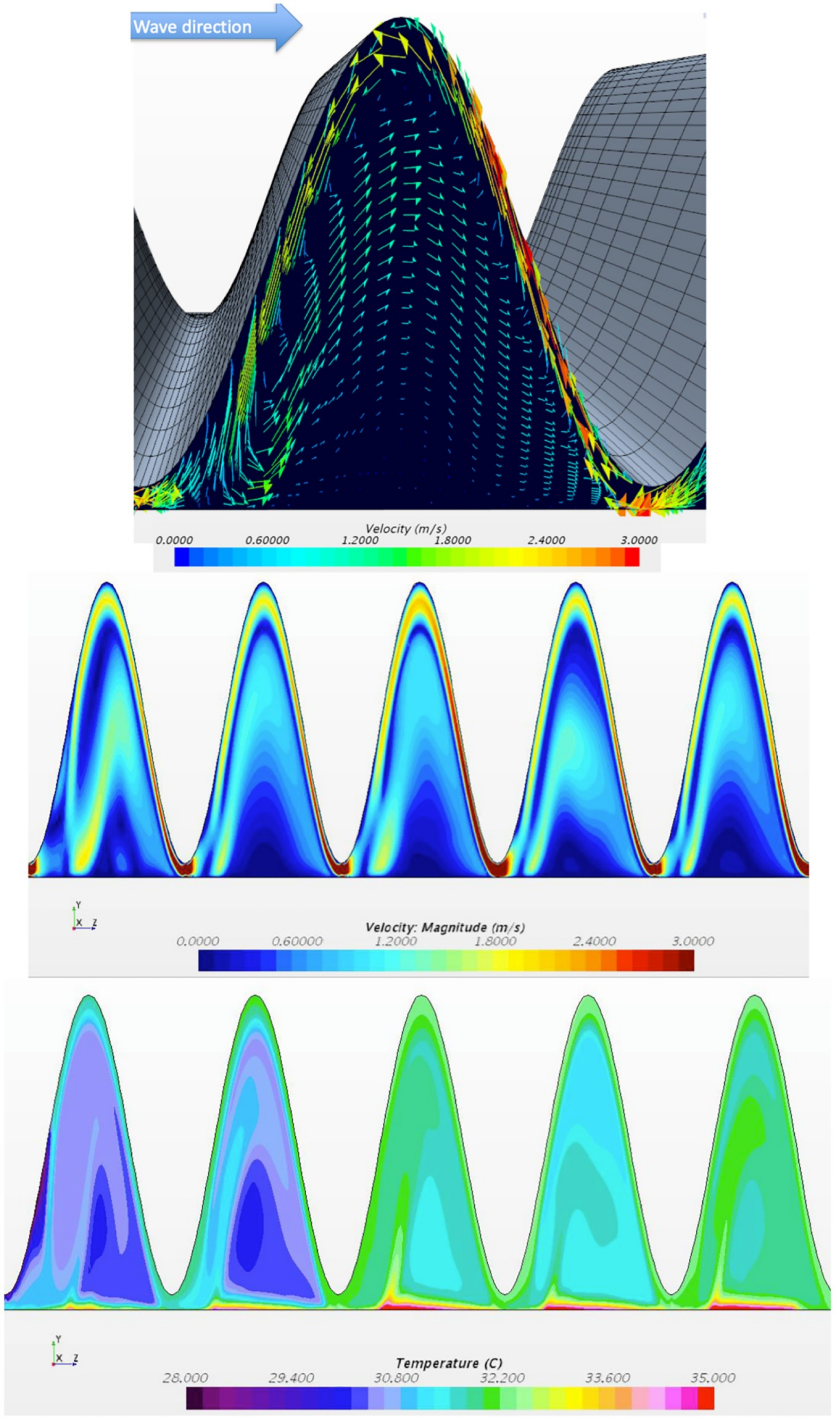
Local analysis. Instantaneous fields: (a) velocity field in a given corrugation, (b) velocity and (c) temperature fields in the symmetry plane. Δ*P*_*s*_ = −50*Pa*, *f*_*r*_ = 50*Hz* and *A* = 90%.

Fig 3 shows fluid behavior (velocity and temperature) in the symmetry plane of the device for an amplitude of 90% and a prescribed pressure difference of -50 Pa. Several points can be drawn from these images. First, the movement of the upper wall of the heat exchanger causes a mixing intensification. In fact, the upper wall pushes the fluid toward the heated sole at the constrictions while in the pockets the vertical wall movement produces a vortex-like effect, which thus enhances mixing. A local jet flowing in the global reverse flow direction can be observed near the membrane due to the combined effect of the dynamic wall and the imposed pressure difference between the outlet and the inlet. The rest of the fluid moves globally with the wave pocket that is pushed in the global desired flow direction. Thus, it flows in the opposite direction of the local jet and the combination of these two phenomena forms the vortex-like process in the pocket (Fig 3a and 3b).

On temperature field (Fig 3c) the thermal boundary layer disruption is clearly visible with cold fluid pushed down toward the sole while it develops partially in the pockets, leading to the heat transfer intensification. As most of the heat transfer occurs where the thermal resistances are the lowest, i.e., in the most constricted portions of the channel, the heat transfer coefficient is thus no longer controlled by the average height of the channel but mainly by its smallest height. This induces remarkably high values of the global heat transfer coefficient, which are not directly governed by the flow rate but predominately by the local wall displacement dynamic.

**Fig 4:**
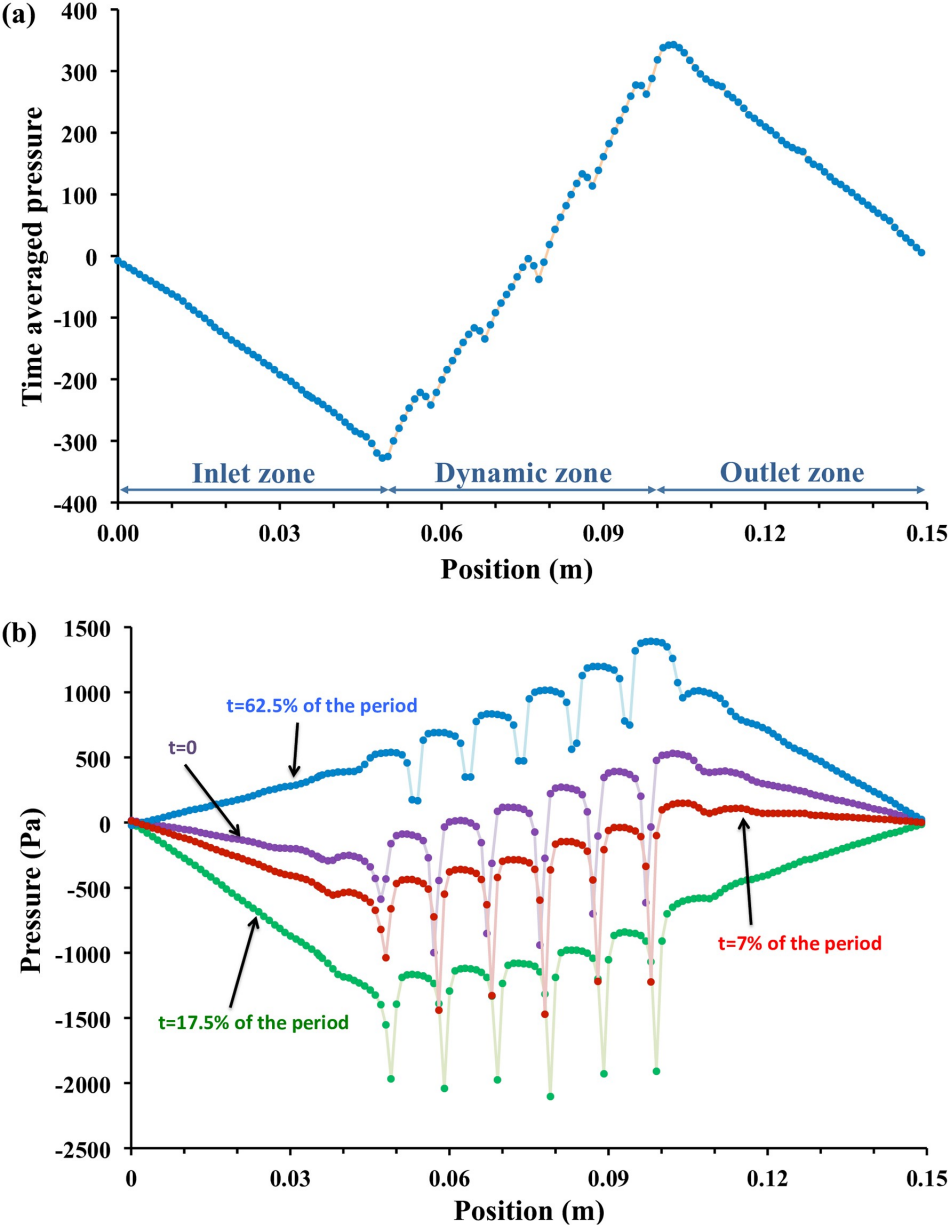
Pressure profiles. (a) Time averaged pressure profile along the main flow axis (b) Instantaneous profiles at various points of time. The dips on the profile correspond to the constriction zone. Imposed pressure difference Δ*P*_*s*_ = 0*Pa*, *f*_*r*_ = 50*Hz* and *A* = 90%.

On Fig 4, both time-averaged and instantaneous pressure profiles along the main flow axis are presented for a zero-pressure difference. Time averaged pressure decreases in inlet and outlet zone as expected while in the dynamic zone the fluid is pumped such as compensating these pressure losses and produce a net flow rate. A rising pressure profile, roughly linear, is obtained. Instantaneous profiles reveal a more complex behavior.

Three main points may be highlighted:

- First, the slope of the static inlet and outlet zones can be either positive or negative depending if the fluid accelerates or decelerates at this time (see also animation provided in S1 Video).
- Secondly, sharp dips are visible, highlighting acceleration effects in the vicinity of constricted zones. From various cases analysis, these effects were found to vary with deformation amplitude. At low amplitude, the local acceleration is not so important due to the distance between membrane and bottom sole, and dip are not marked. At extremely high amplitude (> 95% here), the flow below the constriction becomes negligible and dips are also not important.
- Finally, the profile is stair-like in the actuated zone showing that each corrugation “transports” the fluid (the pressure is nearly constant in a single corrugation) and that the pressure increase is located at the boundary from a corrugation to the following one. Also, the global slope in the actuated zone does not vary significantly with time.

However, the imposed pressure differences lead to different behaviors regarding the pumping effect in the channel. For instance, at low pressure differences between outlet and inlet (−500*Pa* < Δ*P*_*s*_ < −50*Pa*), the system is capable of pumping the fluid due to enough deformation of the wall which in turn saves the viscous loss in the active part of the channel leading to low pressure drop (see Table 3 and following sections for detailed analysis). On the other hand, for high absolute values of the pressure difference (−10000*Pa* < Δ*P*_*s*_ < −1000*Pa*), the system pumps the fluid within the active zone but doesn’t allow compensating the viscous losses due to the flow induced by the prescribed pressure difference (pressure at the outlet is then less than pressure at the inlet), as detailed in the following paragraphs.

**Table 3:** Performances of the dynamic corrugated heat exchanger for an amplitude of 89%.

Pressure difference Δ*P*_*s*_ (Pa)	Heat transfer coefficient (*W*.*m*^−2^.*K*^−1^)	Mass flow rate (*g*.*s*^−1^)	Pressure gain Δ*P*_*g*_ (Pa)	Pumping power ${\dot{W}}_{pp}$ (W)	Pressure work ${\dot{W}}_{mp}$ (W)	Viscous losses ${\dot{W}}_{f}$ (W)	Equivalent pressure drop (Pa)	Merit factor (j/f) (-)
-50	8106	23.7	588.63	1.40 10^−2^	2.63 10^−2^	1.24 10^−2^	522.2	7.41 10^−2^
-100	8109	23.7	546.51	1.30 10^−2^	2.52 10^−2^	1.23 10^−2^	515.5	7.53 10^−2^
-500	8156	24.5	208.99	5.12 10^−3^	1.65 10^−2^	1.14 10^−2^	465.7	8.64 10^−2^
-1000	8499	25.5	-196.27	−5.01 10^−3^	6.14 10^−3^	1.11 10^−2^	436.8	1.00 10^−1^
-5000	11436	30.9	-3677.49	−1.14 10^−1^	−7.81 10^−2^	3.57 10^−2^	1153.8	6.18 10^−2^
-10000	13485	35.8	-8087.27	−2.90 10^−1^	−1.81 10^−1^	1.09 10^−1^	3036.4	3.21 10^−2^

Presentation of various global heat transfer and flow properties. Total work, pressure work, pumping power, viscous loss, equivalent pressure drop, and merit factor are also presented.

## Systematic study

Conventional corrugated channels (heat exchangers) need an external pump for the fluid to flow in the inlet-to-outlet direction of the channel. The main part of the pressure losses is then classically generated by the passage of the flow in the channel. To limit such pressure losses, the dynamic corrugated channel can be used as an aid to the external pump. In the present study, the goal is to compare the thermo-hydraulic performance of the new dynamic corrugated exchanger to classic ones.

The dynamic of the wall, its amplitude of deformation as well as the negative pressure difference imposed are coupled with both heat transfer coefficient and mass flow rate. A separate systematic analysis in terms of mass flow and heat transfer coefficient are presented below.

### Effect of the amplitude and Δ*P*_*s*_ on the flow rate and the heat transfer coefficient

**Fig 5:**
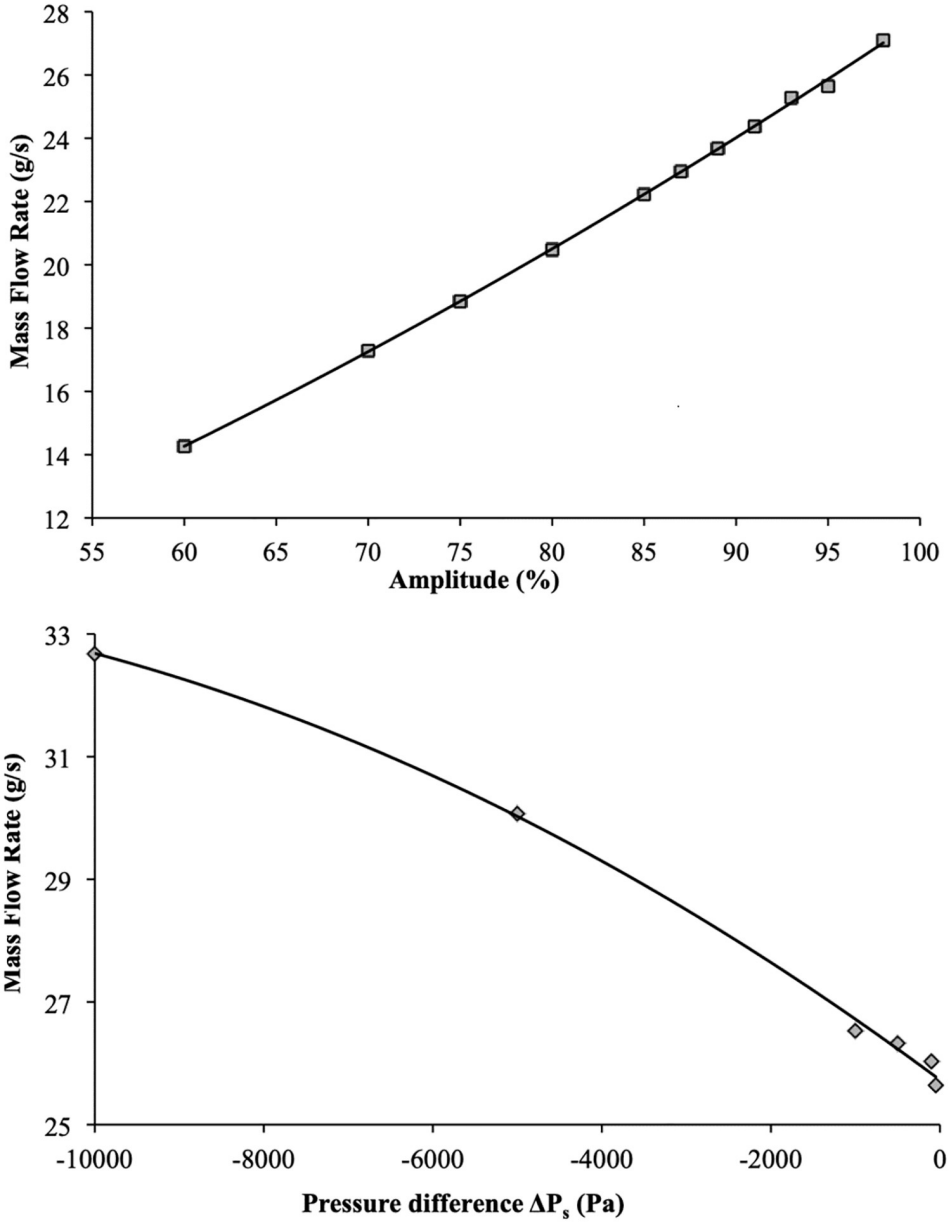
Systematic study. (a) Mass flow rate as a function of relative amplitude when Δ*P*_*s*_ = −50*Pa*, (b) Variation in mass flow rate with imposed pressure difference for *A* = 95%, *f*_*r*_ = 50*Hz*.

As expected for a given pressure difference of Δ*P*_*s*_ = −50*Pa* the mass flow rate depends primarily on the amplitude of deformation imposed on the dynamic wall. Increasing amplitude thus leads to a proportional increase in the mass flow rate (see Fig 5a).

The maximum flow rate can easily be calculated considering that, for high amplitudes, it tends to be controlled by the travelling wave celerity, i.e., wavelength multiplied by frequency. Indeed, when the amplitude is close to 100%, no flow occurs from a pocket to the other. The liquid flows thus at almost the same velocity than the one of the travelling waves. As expected the mass flow rate increases with the increase of the absolute value of pressure difference for a given amplitude (Fig 5b).

**Fig 6:**
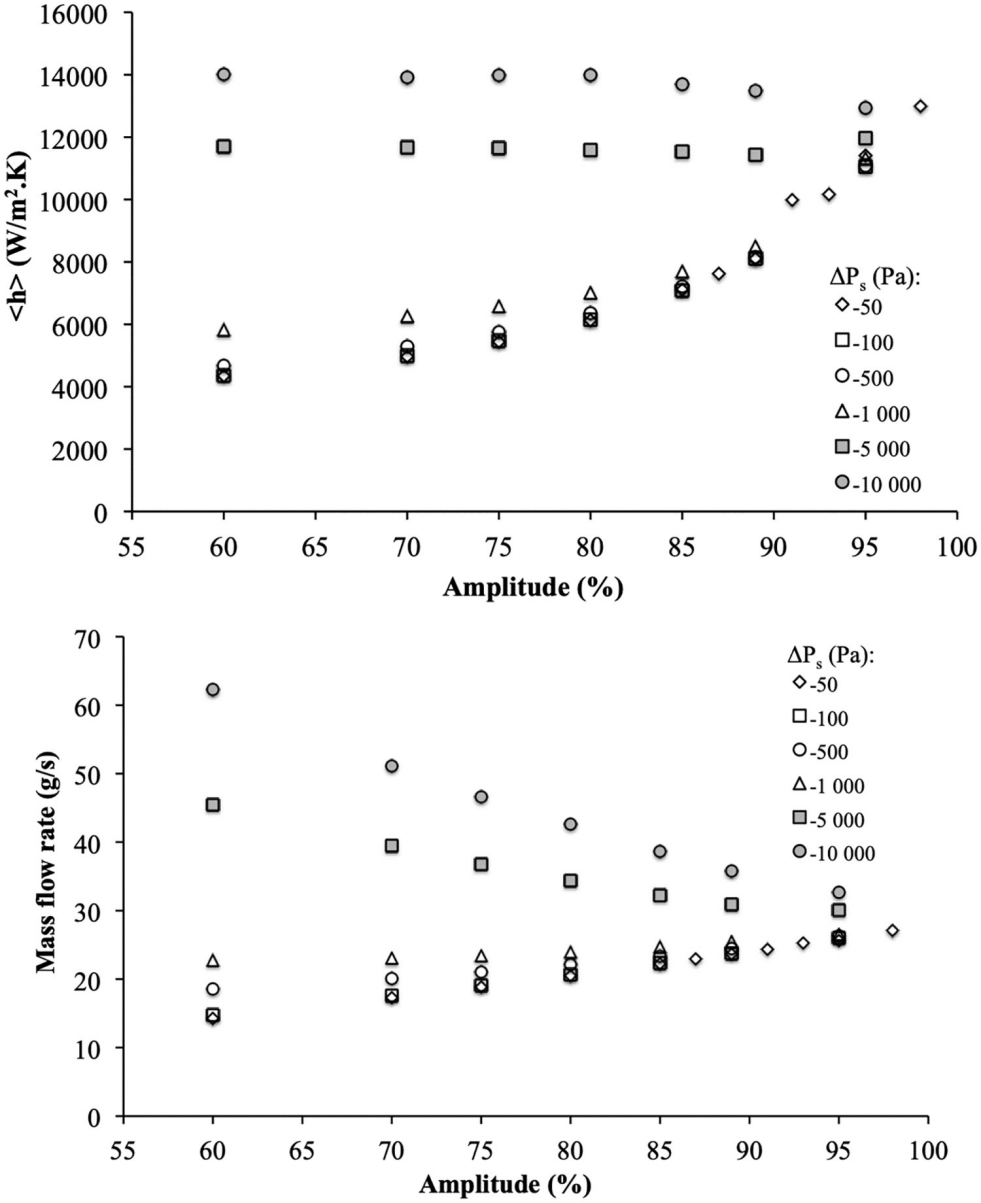
Global analysis. (a) Global heat transfer coefficient (< *h* >), and (b) Mass flow rate as a function of relative amplitude for different Δ*P*_*s*_ and *f*_*r*_ = 50*Hz*.

Thermo-hydraulic characteristics in terms of mass flow and heat transfer coefficient are presented as a function of the amplitude for a set of the imposed pressure difference between the inlet and outlet of the channel in Fig 6. It is noticeable that for relatively small absolute values of the pressure difference (−1000*Pa* ≤ Δ*P*_*s*_ ≤ −50*Pa*) in the heat exchanger, the mass flow rate and heat transfer coefficient follow the same tendency as shown in [15] and [16]. The flow is mainly driven by the actuation of the wall. In that case the mass flow rate increases with the relative amplitude of the wave to reach the volumetric flow rate of the wave displacement at 100% amplitude. The heat exchanger involves thus high mass flow rate for high amplitudes and simultaneously high heat transfer coefficient was obtained. For a given frequency (*f*_*r*_ = 50*Hz*, in the present study), the dynamic wall drives the fluid in the same direction that the movement of the sine wave. At high amplitudes (*A* > 85%), fluid pockets are formed between each sine wave, which makes the flow reaching the value of the maximum flow rate, the system tending toward a peristaltic pump behavior. The heat transfer coefficient increases for high amplitudes because of the recirculation and acceleration of fluid exerted in and between the pockets. Moreover, this heat transfer coefficient can be particularly high just upstream and/or downstream the minimum thickness zone, when cold fluid is brought toward the solid wall by the “jet” effect induced by the complex flow pattern.

For higher absolute value of the pressure-differences (Δ*P*_*s*_ ≤ −5000*Pa*), the system is no more able to compensate all the internal viscous losses. Compared to the value for moderate pressure difference, the variation in the heat transfer coefficient for the highest pressure-difference is about 13% for A = 95%. The heat transfer coefficient still reaches a high value even for lower mass flow rate at high pressure-difference (see Fig 6a and 6b for instance for Δ*P*_*s*_ = −10000*Pa* and *A* = 95%). So, when the amplitude increases (*A* > 90%), the mass flow rate and heat transfer coefficient tend to reach constant values whatever is the pressure difference.

### Comparison with straight channel

**Fig 7:**
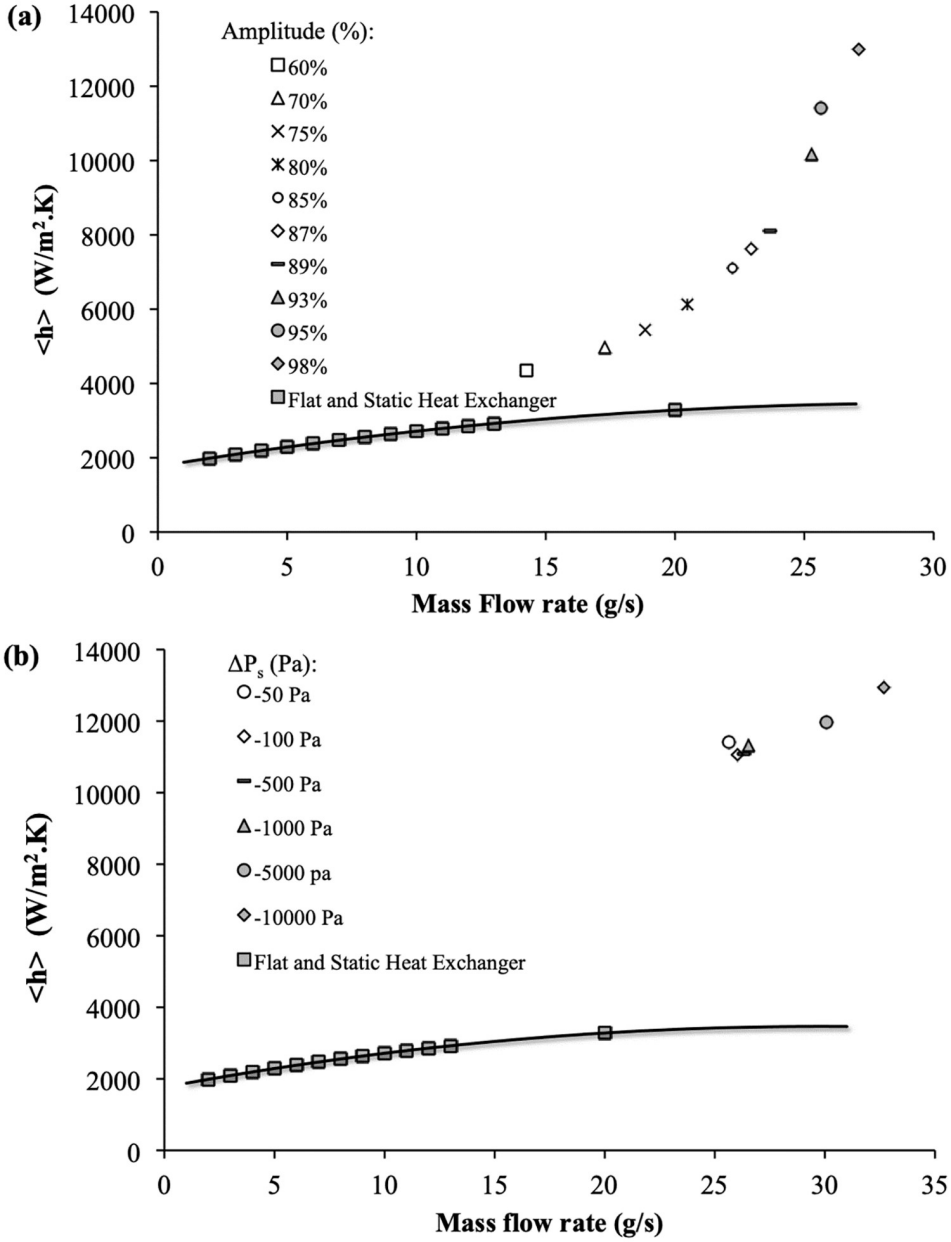
Global heat transfer coefficient. < *h* > as a function of mass flow rate for (a) different relative amplitudes and Δ*P*_*s*_ = −50*Pa*, and for (b) different Δ*P*_*s*_ for a relative amplitude of 95% for the dynamic corrugated channel. *f*_*r*_ = 50*Hz*. Comparison with flat and static channel is also presented.

In order to compare the thermo-hydraulic behavior of the dynamic heat exchanger to a heat exchanger made of a straight channel, the heat transfer coefficient is reported as a function of the mass flow rate for various amplitude on Fig 7a. and for various Δ*P*_*s*_ on Fig 7b.

As the flow rate, the heat transfer coefficient also depends on the amplitude for a given average channel height. The higher the amplitude, the more significant the heat transfer intensification is, compared to cases without wall deformation, i.e., straight channel, as presented in Fig 7a. Moreover, at very high relative amplitude and for moderate absolute values of the pressure differences (−1000 ≤ Δ*P*_*s*_ ≤ 0*Pa*), the heat transfer coefficient does not vary (as well as the mass flow rate), as shown in Fig 7b. The following considerations can explain these different observations:

- The dynamic corrugation of the upper wall of the heat exchanger causes a mixing intensification and therefore a reduction in the establishment lengths compared to a straight channel.
- Majority of the heat transfer occurs where the thermal resistances are lowest, i.e., at the maximal constrictions where the cross section for the flow is lowest in the channel and for very high amplitudes. As already mentioned, the value of the heat transfer coefficient is thus no longer controlled by the average size of the channel, but by the smallest dimension of flow passage in the channel, leading to very high values of the heat transfer coefficient.
- For high amplitude and moderate pressure difference, the system acts as a nearly volumetric pump and compensates its own viscous losses. The mass flow rate, and consequently the heat transfer coefficient, is then relatively independent from the pressure difference, this latter being much lower than the pressure variation within the actuated channel.

To summarize, remembering that the average hydraulic diameter is constant, the velocity of the fluid is less than the wave velocity for low pressure-difference. In this case the wave displacement contributes positively to the entrainment of the fluid. The fluid velocity increases with the pressure difference and becomes higher than the wave one. In this case, the channel induces a hydraulic resistance, which increases with the amplitude. Thus, for a given pressure difference the mass flow rate decreases. The higher the fluid velocity, the closer the situation to static corrugated channel case. The enhancement due to actuation becomes then limited. As discussed in the following section, this may not justify anymore the added expense of energy to obtain a dynamic corrugated wall.

## Performances

The viscous dissipation can be defined in an analog way than in [16] by:

$$\begin{array}{c} {\dot{W}}_{f}={\dot{W}}_{mp}-{\dot{W}}_{pp}=\int_{S_{dw}}(-P\bar{\bar{I}}\cdot \vec{n})\cdot \vec{u}\;ds-Q_{v}\Delta P_{g} \end{array}$$ (3)

where the first term is the mechanical power and the second one represents the pumping power.

**Fig 8:**
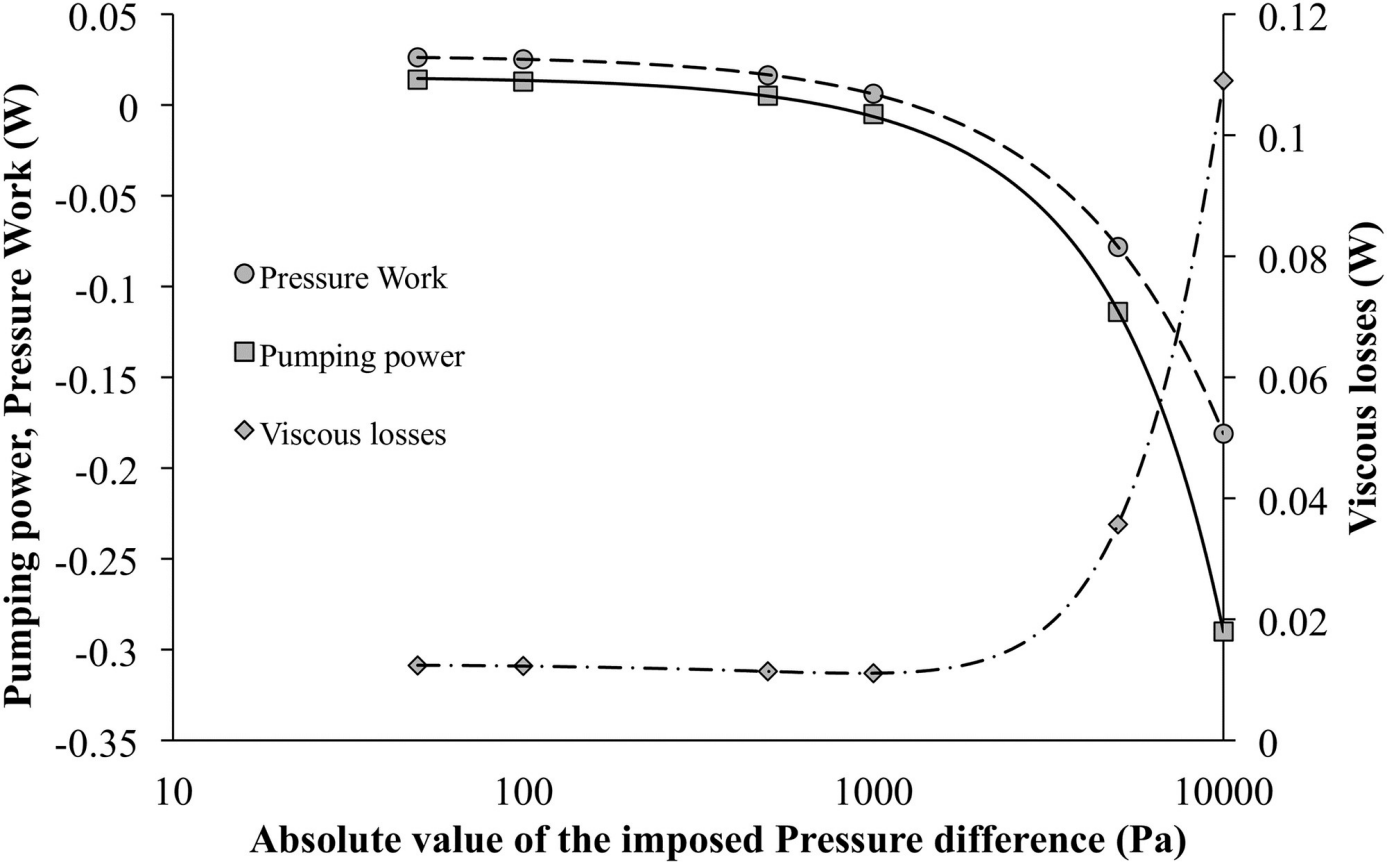
Performances of the dynamic corrugated heat exchanger. Pumping power, pressure work and viscous loss for corrugated moving channel as a function of the imposed negative pressure differences for relative amplitude of *A* = 89% and *f*_*r*_ = 50*Hz*.

Increasing the prescribed pressure difference for a given amplitude leads to a decrease of both mechanical power applied on the actuated surface (${\dot{W}}_{mp}$) and pumping power (${\dot{W}}_{pp}$) as shown in Table 3. On the other hand, viscous dissipation (${\dot{W}}_{f}$) is slightly decreasing when |Δ*P*_*s*_| < 1000*Pa* and then increase (Fig 8).

**Fig 9:**
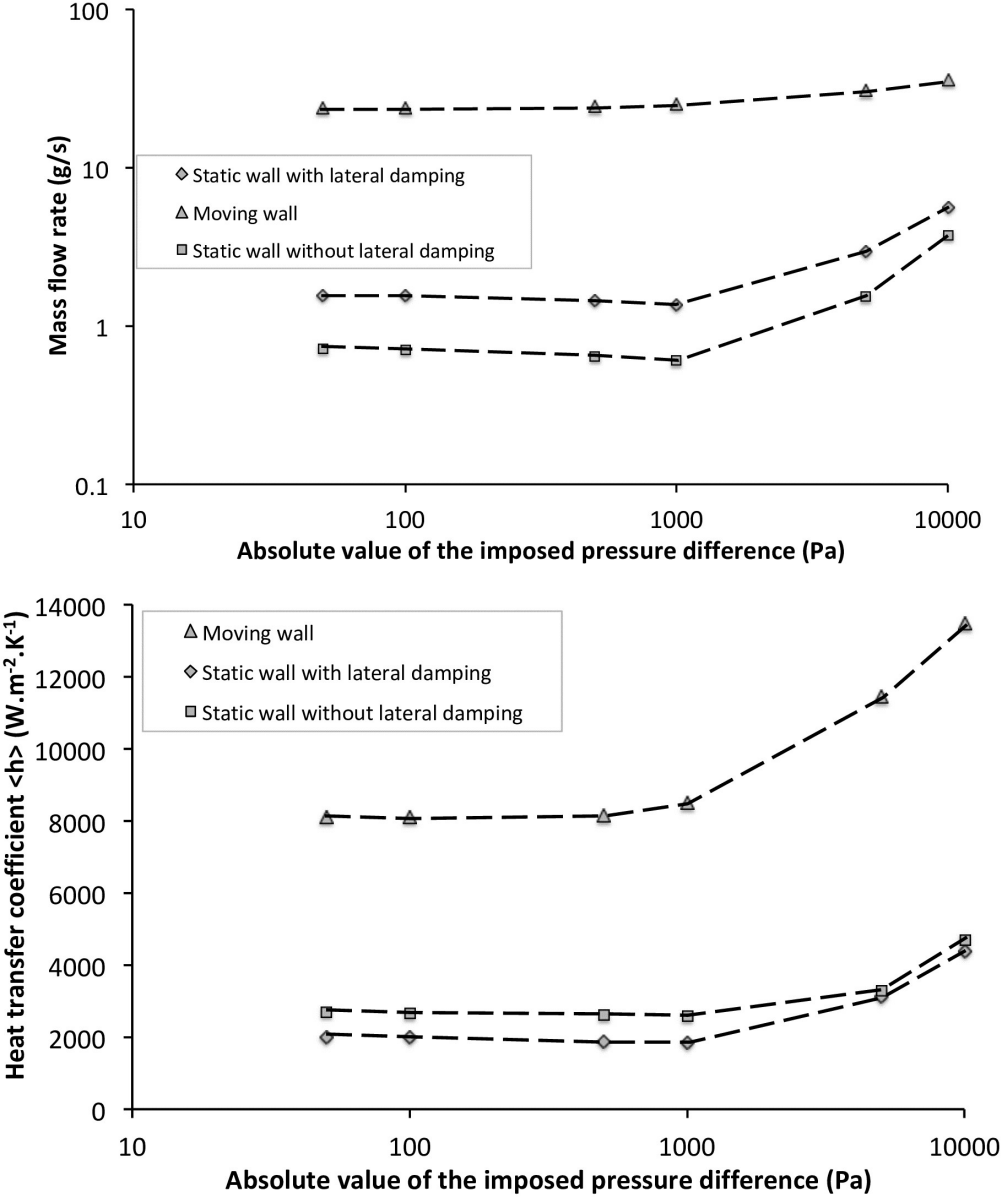
Performances of the dynamic corrugated heat exchanger. (a) Mass flow rate and (b) Heat transfer coefficient for corrugated moving, static with and without lateral damping (log-log scales). Different imposed negative pressure differences for relative amplitude of *A* = 89% and *f*_*r*_ = 50*Hz*.

In fact, looking at the Fig 9a, it can be seen that there is a transition zone in the mass flow rate behavior between the imposed pressure differences of 1000 Pa and 5000 Pa.

For the lower pressure differences, the mass flow rate is increasing with amplitudes and then the heat exchanger can be considered as a pumping system, while it is decreasing for the higher pressure-differences. Negative value of pumping power (see Table 3) can be termed as “pumping losses” and such a heat exchanger induces a flow resistance in the hydraulic circuit. This effect is easily observable on the resulting viscous dissipation with a transition region between 1000 Pa and 5000 Pa.

Heat transfer coefficients and mass flow rates were first calculated for static corrugated channel with and without lateral damping (*Y*_1_ = 0 in case of no lateral damping, equation A-1) for the same amplitude of 89% by applying same equivalent pressure drops in order to compare the performance with our proposed exchanger (Table 4). Static corrugated wall without any lateral damping is a one-dimensional case.

**Table 4:** Performances of the static corrugated heat exchanger with an amplitude of 89%.

Both channels		Static corrugated channel with lateral damping			Static corrugated channel without lateral damping		
Pressure difference Δ*P*_*s*_ (Pa)	Equivalent pressure drop Δ*P*_*d*_ (Pa)	Heat transfer coefficient (*W*.*m*^−2^.*K*^−1^)	Mass flow rate (*g*.*s*^−1^)	Merit factor (j/f) (-)	Heat transfer coefficient (*W*.*m*^−2^.*K*^−1^)	Mass flow rate (*g*.*s*^−1^)	Merit factor (j/f) (-)
-50	522.16	2013	1.56	1.22 10^−3^	2688	0.712	7.39 10^−4^
-100	515.54	2000	1.55	1.21 10^−3^	2681	0.704	7.38 10^−4^
-500	465.70	1896	1.43	1.17 10^−3^	2631	0.637	7.25 10^−4^
-1000	436.76	1834	1.36	1.15 10^−3^	2601	0.598	7.18 10^−4^
-5000	1153.75	3139	2.95	1.62 10^−3^	3291	1.53	8.81 10^−4^
-10000	3036.40	4403	5.64	1.65 10^−3^	4684	3.70	1.15 10^−3^

Presentation of various global heat transfer, flow properties and merit factor.

We compared the mass flow rates and heat transfer coefficients obtained with static and dynamic heat exchangers on Fig 9a and 9b.

Of course, for the static case these two quantities are proportional and increase with pressure difference. The behavior of mass flow rate and heat transfer coefficient in case of dynamic corrugated exchanger is approximately identical between low and moderate pressure differences (1000*Pa* > |Δ*P*_*s*_| > 50*Pa*) while there is an increase in mass flow rate as well as in heat transfer coefficient values at higher absolute values of pressure difference (10000*Pa* > |Δ*P*_*s*_| > 5000*Pa*). Compared to static corrugated channel case, proposed dynamic exchanger has the same behavior but with higher mass flow rate (up to a factor of 33) and heat transfer coefficient (up to a factor 4) for different values of pressure drop.

The merit factor is classically defined as the ratio between the Colburn factor (j) and the friction factor (f). A surface having a higher j/f factor is “good” because it requires lower free flow area and hence a lower frontal area for heat exchanger [21]. In the same way than in [16] we use Eq 4 to define the equivalent pressure drop (Δ*P*_*d*_) due to friction in active devices:

$$\begin{array}{c} \Delta P_{d}={\dot{W}}_{f}/Q_{v} \end{array}$$ (4)

As expected, this quantity follows the same trend than viscous dissipations power. It is slightly decreasing for imposed pressure difference lower than 1000 Pa but increasing after (Table 3). The performance of heat exchangers can be evaluated as follows:

$$\begin{array}{c} \frac{j}{f}=\frac{{L\rho <\|u\|}^{2}>}{2|\Delta P_{d}|D_{h}}\frac{<Nu>}{<Re>Pr^{1/3}} \end{array}$$ (5)

**Fig 10:**
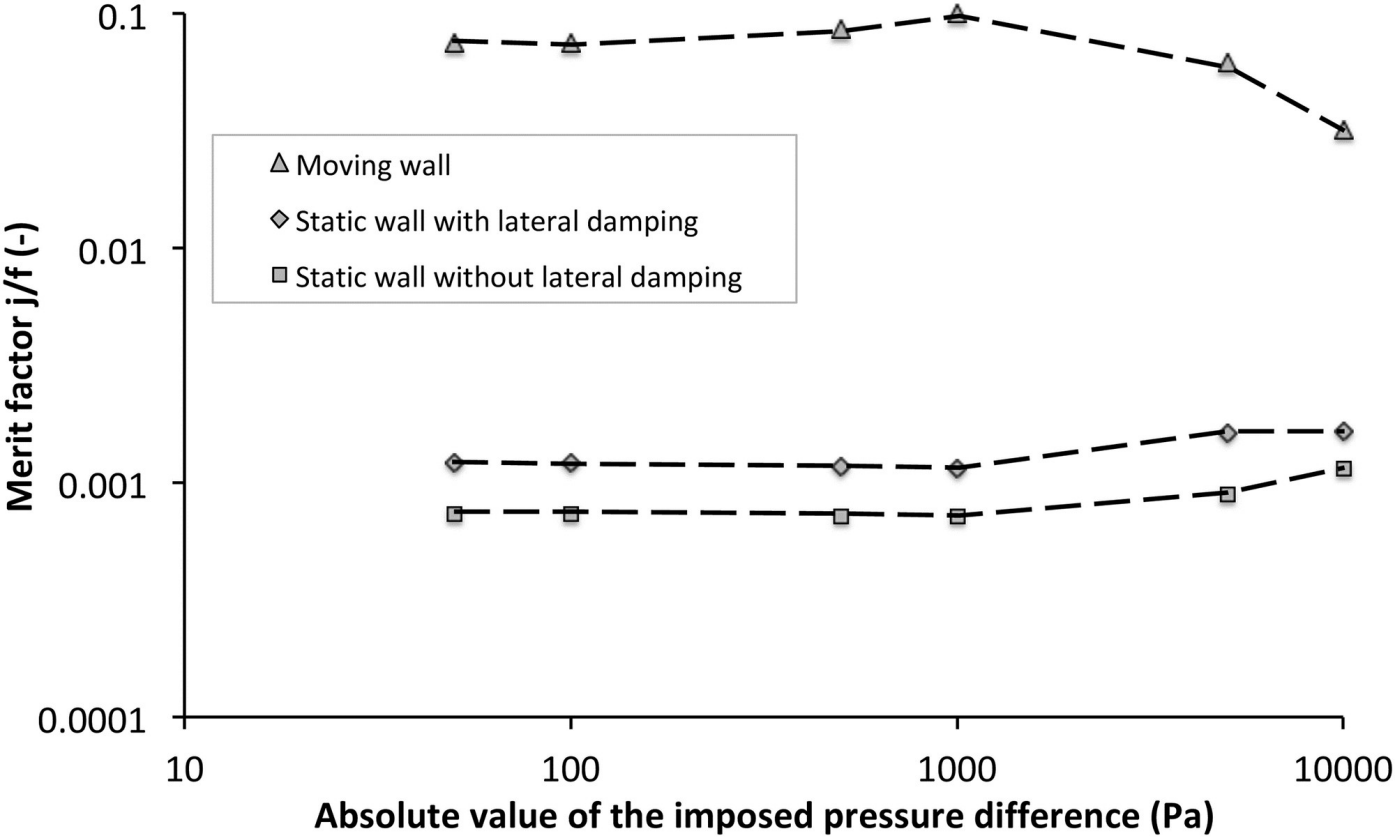
Performances of the dynamic corrugated heat exchanger. Merit factor for corrugated moving, static with and without lateral damping (log-log scales). Different imposed negative pressure differences for relative amplitude of *A* = 89% and *f*_*r*_ = 50*Hz*.

Merit factors (j/f) are presented in Fig 10 as well as Tables 3 and 4 for both static and dynamic heat exchangers.

Indeed, for classical static corrugated channel, performance increases slightly with flow rate. For low pressure-difference (|Δ*P*_*s*_| < 1000*Pa*) the performances of dynamic heat exchanger do not vary (*j*/*f* ≈ 0.1). Indeed mass flow rates and heat transfer coefficients are almost constant for such conditions. On the other hand, for |Δ*P*_*s*_| > 1000*Pa*, even if heat transfer coefficients are increasing, the viscous dissipations also increase significantly and thus, the performance of the dynamic heat exchanger decrease with imposed pressure differences. When the prescribed pressure difference increases, the flow rate increases proportionally and the part due to the dynamic pumping effect is reduced. The behavior of the system is thus progressively closer to that of a passive system and its efficiency is reduced. More precisely, the enhancement compared to the static case is close to 100 for low flow rate (|Δ*P*_*s*_| < 1000*Pa*) then decreases down to 20 for higher ones (|Δ*P*_*s*_| = 10000*Pa*).

## Conclusion

In order to improve the performance of thermal systems in low Reynolds number range, an innovative concept of heat exchanger at millimeter scale is proposed. It consists in dynamically deforming one of the walls that lower down the pressure drop as compared to various existing systems at miniature scale.

Single-phase flow performance enhancement in terms of heat transfer and mass flow rate as function of prescribed pressure difference were studied. Both heat transfer coefficient and mass flow rate increase with actuation amplitude at given pressure difference across the channel. The actuated zone in the heat exchanger exhibits two behaviors: it allows the fluid to flow by its own virtue -i.e. it compensates the fluid viscous losses- while sometimes it acts as the hydraulic resistance. The relative values of fluid and traveling wave velocities clearly govern the device behavior. The behavioral transitions occur at a critical pressure difference. Though the performance of the proposed system declines at high negative pressure difference, it remains very high as compared to conventional corrugated channels (enhancement factor varying from 100 down to 20 with increasing prescribed pressure difference).

## Supporting information

S1 Video Illustration of the mixing effect in the actuated channel. Virtual particles are transported in the flow. The color scale represents the temperature of the particles. https://amubox.univ-amu.fr/s/video.
(M4V)

S1 Appendix Management of dynamic corrugated wall. (PDF)
