# Supplementary material for: Heat transfer intensification in an actuated heat exchanger submitted to an imposed pressure drop
Source: PLoS One. 2019 Jul 11;14(7):e0219441. doi: 10.1371/journal.pone.0219441 (PMC6622530; doi:10.1371/journal.pone.0219441)
Supplement: S1 Appendix — (PDF) [file pone.0219441.s002.pdf]

The displacement of the membrane is defined by:

$$y(z,t) = \delta(1 + AY_1Y_2sin(2\pi(f_rt + \omega z)))$$

where δ is a constant displacement used to control the channel height, A is the relative amplitude of the displacement, Y_1 and Y_2 are two damping functions that take into account the effects of the boundaries of the actuated zone, f_r is the frequency, t is the time and ω is the number of waves per unit length, respectively.

The lateral damping is managed as follows:

$$\text{if } x > 0.002m : Y_1 = 1 \text{ else: } Y_1 = (x/0.0015)^3$$

For the longitudinal damping we used:

$$\begin{aligned} \text{if } z < 0.05m \ &: Y_2 = (z/0.05)^4 \\ \text{if } z > 0.05m \text{ and if } z < 0.10m &: Y_2 = 1 \\ \text{if } z > 0.10m \ &: Y_2 = (1 - (z - 0.10)/0.05)^4 \end{aligned}$$
